# Comprehensive analysis of the potential biological significance of cuproptosis-related gene LIPT2 in pan-cancer prognosis and immunotherapy

**DOI:** 10.1038/s41598-023-50039-x

**Published:** 2023-12-21

**Authors:** Wangbiao Wang, Shiang Li, Yumian Huang, Jun Guo, Lili Sun, Gang Sun

**Affiliations:** Department of Breast and Thyroid Surgery, The Affiliated Cancer Hospital of Xinjiang Medical University, No. 789 Suzhou East Street, Urumqi, 830011 Xinjiang People’s Republic of China

**Keywords:** Cancer, Computational biology and bioinformatics, Genetics, Immunology, Molecular biology, Biomarkers, Medical research, Oncology

## Abstract

Lipoyltransferase 2 (LIPT2) acts as a key enzyme involved in fatty acid metabolism and cell membrane synthesis. However, the biological function of LIPT2 in various cancer types and its potential significance in prognosis continue to be unresolved. For this analysis, we evaluated the expression levels and the significance of prognosis of LIPT2 gene in all cancers by various bioinformatics methods. The results found that LIPT2 was dramatically overexpressed in the vast majority of cancers. The upregulated LIPT2 was related to bad prognosis in Brain Lower Grade Glioma (LGG), Glioma (GBMLGG), Glioblastoma multiforme (GBM), Kidney Chromophobe (KICH), and High-Risk Wilms Tumor (WT), while it had a favorable prognosis in Kidney renal clear cell carcinoma (KIRC), and Ovarian serous cystadenocarcinoma (OV), Pan-kidney cohort (KIPAN). Furthermore, we assessed the mutation status, methylation levels, and immune status of LIPT2 in pan-cancer. Single-cell sequencing results revealed the correlation of LIPT2 expression with various biological characteristics such as DNA lesion, tumor angiogenesis, cell apoptosis, metastasis, and invasion. Enrichment analysis unveiled potential molecular regulatory mechanisms. In conclusion, our research reveals a detailed key role of LIPT2 in the progression, prognosis, and immune efficacy of various forms of cancer. Therefore, we have reason to believe that LIPT2 has the potential to be a candidate biomarker for tumors.

## Introduction

Cancer is a serious disease that poses a significant burden on human health and socio-economic factors^1^. According to statistics, about 19 million people worldwide are confirmed with cancer each year, with approximately 10 million deaths attributed to cancer^2^. Cancer has become one of the leading causes of mortality globally^3^. Therefore, understanding and identifying valuable broad-spectrum cancer genes is crucial in revealing potential mechanisms underlying the formation and evolution of different tumors.

Lipoyltransferase 2 (LIPT2) is an enzyme widely present in the cytoplasm, involved in the acyl transfer reaction during fatty acid metabolism^4–6^. Its main function is to combine fatty acids with coenzyme A (CoA) to form acyl-CoA, which is a crucial step in the intracellular metabolism of fatty acids^4,7^. LIPT2 gene mutations may lead to disruption of fatty acid metabolism and promote the occurrence of cancer^8–10^. For example, some studies have shown an increase in LIPT2 gene copy number in patients with head and neck squamous cell carcinoma^9^. In triple-negative breast cancer patients, the overexpression of LIPT2 is related to mutations, and high amplification of LIPT2 is associated with reduced immune infiltration^10^. However, there is currently insufficient research on the potential role of LIPT2 in different types of cancer.

This article explores the possible mechanisms and biological roles of LIPT2 in cancer using various bioinformatics techniques. We compared the level of LIPT2 expression and its consequential contribution to survival in the TCGA and TCGA_GTEx datasets. We also studied its gene mutations, methylation levels, and their relationship with immune response. The results of the analysis demonstrate the biological significance of LIPT2 in pan-cancer and its good predictive role in immunotherapy response. It is expected to provide novel targets as well as strategies for the prognosis and treatment of cancer.

## Results

### Differential expression of LIPT2 in cancers

**Figure 1:**
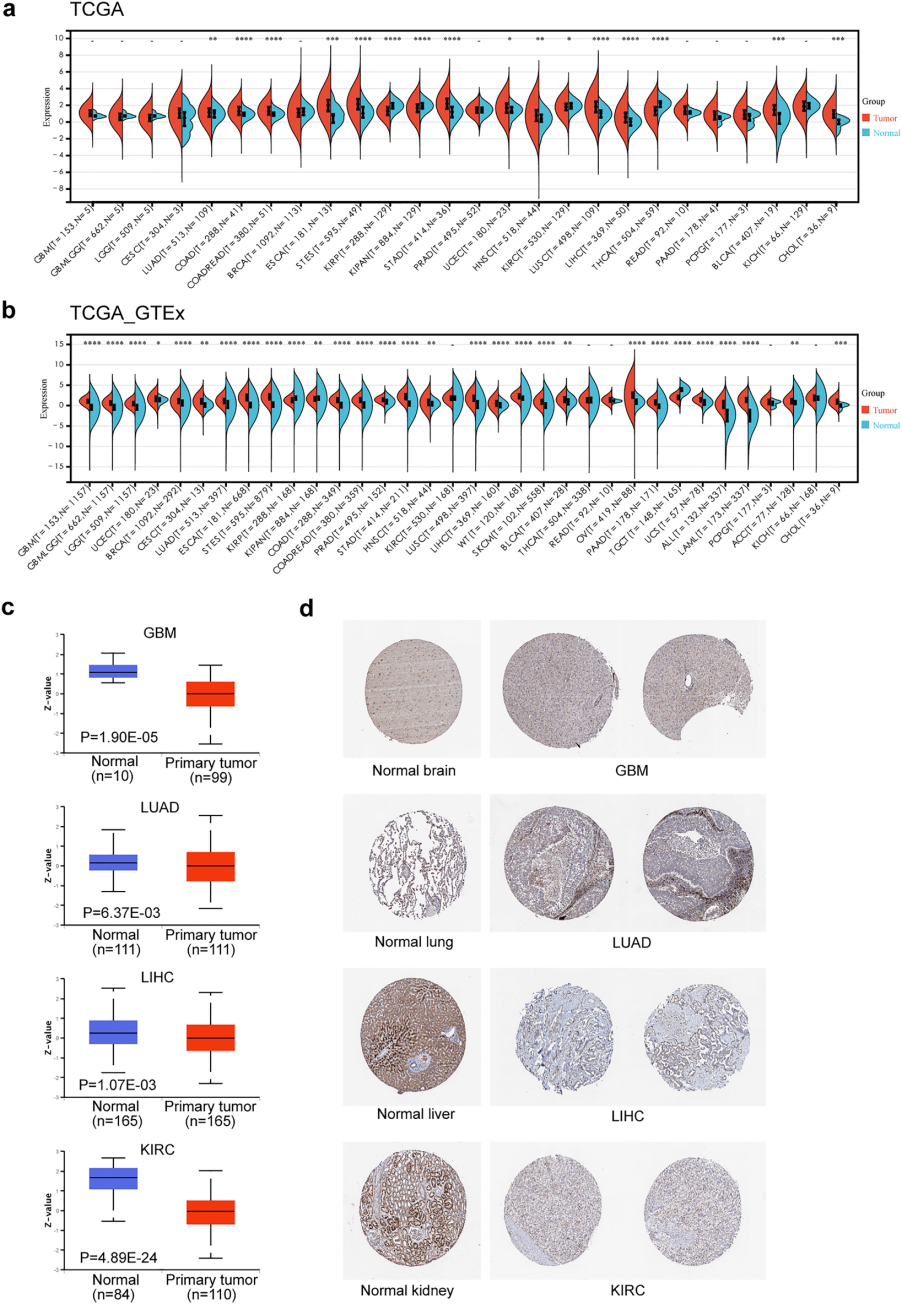
Differential expression of LIPT2 in pan-cancer. (**a,b**) Differential expression of LIPT2 between different tumors and normal tissues, based on TCGA (**a**) and TCGA_GTEx (**b**) data sets, *p < 0.05, **p < 0.01, ****p < 0.001, – not significant. (**c**) CPTAC evaluates the expression levels of LIPT2 total protein in GBM, KIRC, LUAD, and LIHC. (**d**) Representative immunohistochemistry (IHC) images of LIPT2 in GBM, KIRC, LUAD, and LIHC in the HPA database.

First, we investigated the expression abundance of LIPT2 in pan-cancer in the TCGA dataset (Fig. 1a). Since the number of normal tissue samples in TCGA is limited, we conducted additional analysis on the expression variances of LIPT2 via the TCGA_GTEx datasets. The findings revealed that LIPT2 was remarkably up-regulated among 26 tumors, including GBMLGG, significantly down regulated in 3 types of tumors, and no differential expression was found in 5 types of tumors (Fig. 1b). In addition, we found through the GEPIA2 website that LIPT2 expression had an impact on the pathological staging of KIRC, Thyroid carcinoma (THCA), and Liver hepatocellular carcinoma (LIHC) patients (Supplementary Fig. 1a). Following that, we evaluated the protein expression of LIPT2 using the CPTAC dataset. The results showed that total LIPT2 protein was significantly down regulated in GBM, KIRC, Lung adenocarcinoma (LUAD), and LIHC (Fig. 1c). This was consistent with the immunohistochemistry (IHC) results in the HPA database (Fig. 1d). The expression levels of LIPT2 mRNA in healthy human tissues and cancer cell lines were also studied using the HPA database (Supplementary Fig. 1b,d). LIPT2 mRNA was expressed at relatively high levels in most cancer cell lines (Supplementary Fig. 1d), and at low levels in normal human tissues, except in the testis, skeletal muscle, and tongue (Supplementary Fig. 1b). Interestingly, total LIPT2 protein showed moderate or high expression levels in healthy human tissues (Supplementary Fig. 1c). This may be related to post-translational regulation or modification.

### Prognostic value of LIPT2 in pan-cancer

**Figure 2:**
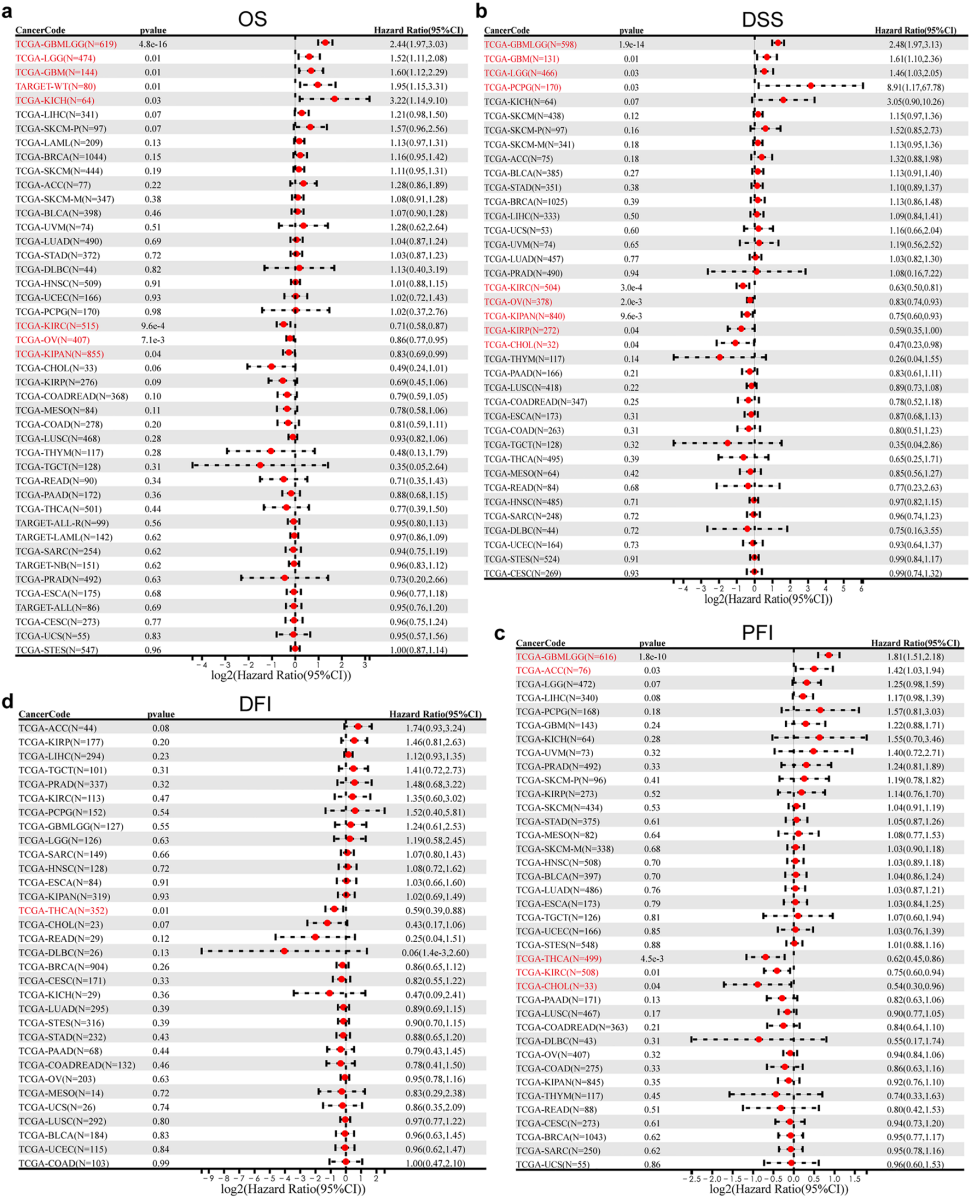
Correlation of LIPT2 expression with prognosis features in pan-cancer patients, including overall survival (OS) (**a**), disease-specific survival (DSS) (**b**), progression-free interval (PFI) (**c**), and disease-free interval (DFI) (**d**).

We analyzed the impact of LIPT2 expression differences on the prognosis of cancer patients. The Cox regression results showed a significant correlation between LIPT2 expression and overall survival (OS) in eight types of tumors (Fig. 2a). Consistent with further Kaplan–Meier survival analysis results, high LIPT2 expression was relevant to unfavorable OS in GBM, GBMLGG, LGG, KICH, and WT, while high expression of LIPT2 showed better OS in KIPAN, KIRC, and OV (Supplementary Fig. 2a). LIPT2 expression was also prominently pertinent to disease-specific survival (DSS) in nine types of tumors (Fig. 2b). In GBM, GBMLGG, LGG, and Pheochromocytoma and Paraganglioma (PCPG), high expression of LIPT2 indicated poor DSS, while low expression of LIPT2 indicated poor DSS in KIPAN, KIRC, Kidney renal papillary cell carcinoma (KIRP), Cholangiocarcinoma (CHOL), and OV (Supplementary Fig. 2b). LIPT2 expression was significantly correlated with progression-free interval (PFI) in five types of tumors (Fig. 2c) and disease-free interval (DFI) in one type of tumor (Fig. 2d). Upregulation of LIPT2 was significantly associated with shorter PFI in GBMLGG and Adrenocortical carcinoma (ACC), and longer PFI in KIRC, THCA, and CHOL. It was also significantly associated with longer DFI in THCA (Supplementary Fig. 3a,b).

Considering the significant correlations between LIPT2 expression and OS, DSS, and PFI in GBMLGG patients, further analysis was conducted. Supplementary Table 1 presents the connection between LIPT2 expression and clinical features of GBMLGG patients. High expression of LIPT2 exhibited a significant association with WHO grade, IDH status, 1p/19q codeletion, gender, age, and histological type (Supplementary Table 1). Receiver operating characteristic (ROC) curve analysis demonstrated that LIPT2 expression had a diagnostic value for GBMLGG, with an area under the curve (AUC) of 0.855, indicating high diagnostic accuracy (Supplementary Fig. 3c). Cox analysis revealed that WHO grade, IDH status, age, and LIPT2 expression were isolated prognostic factors for GBMLGG (Supplementary Table 2). Based on the Cox analysis results, we developed a nomogram model to forecast the 1-year, 3-year, and 5-year OS probabilities of patients and evaluated the predictive accuracy using calibration curves (Supplementary Fig. 3d). The internal validation of the nomogram showed a C-index of 0.853 (0.842–0.863), indicating good predictive ability of LIPT2 for OS in GBMLGG, and the calibration curves also demonstrated high accuracy of the nomogram model (Supplementary Fig. 3e). The findings imply that LIPT2 may be a latent prognostic indicator for various cancers, particularly GBMLGG.

### Mutation analysis of the LIPT2 gene

**Figure 3:**
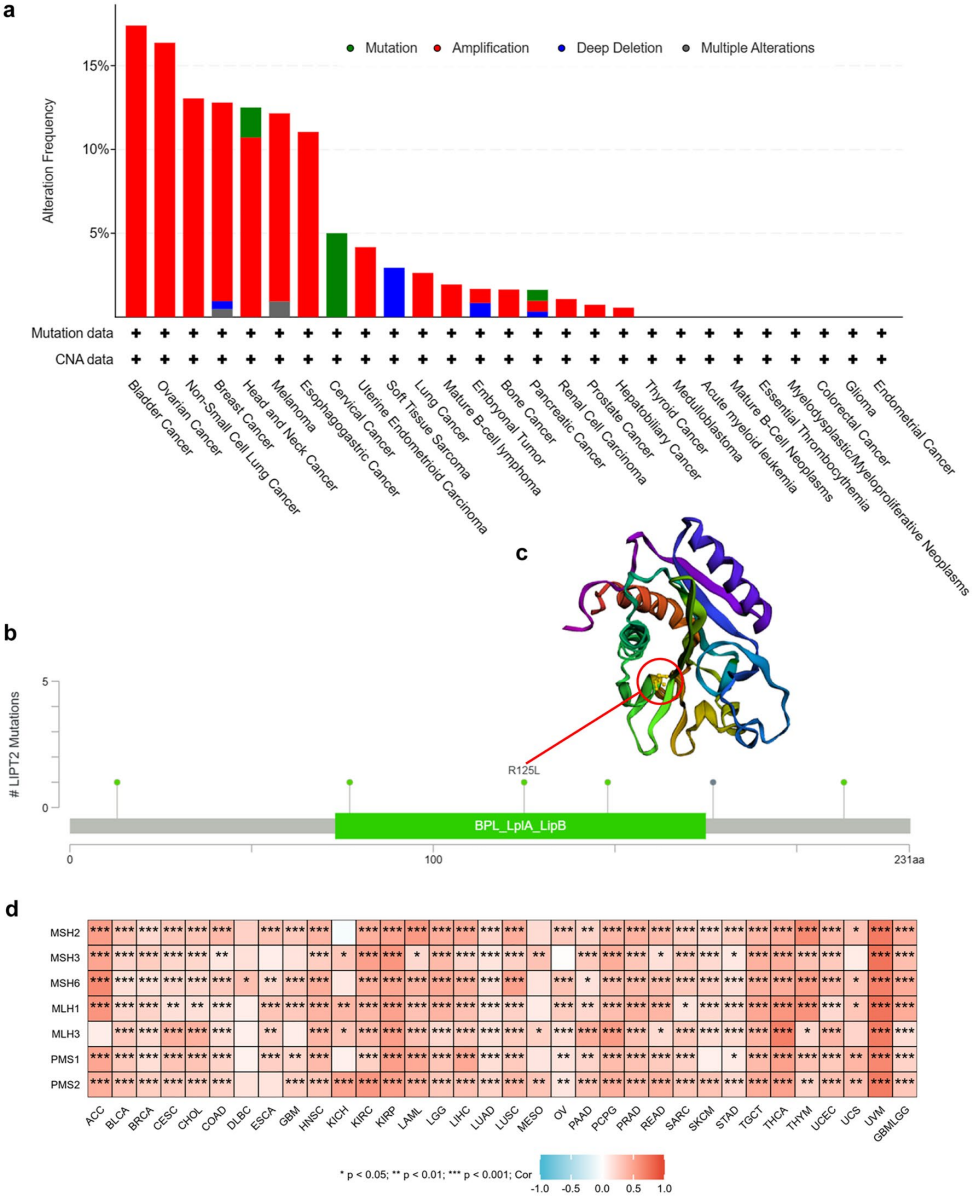
LIPT2 gene mutations in pan-cancer. (**a–c**) cBioPortal shows the mutation types of LIPT2 gene in pan-cancer (**a**), the R125L mutation site (**b**), and the R125L alteration in the 3D structure (**c**). (**d**) Correlation between LIPT2 expression and MMRS gene in pan-cancer.

We assessed the mutations of LIPT2 in pan-cancer using the cBioPortal database. The analysis revealed that LIPT2 amplification was found in more than 7 types of cancers, with Bladder Urothelial Carcinoma (BLCA) having the highest LIPT2 amplification rate (> 15%). The mutation rate in Head and Neck Cancer was approximately 12%, and the deep deletion occurrence rate was highest in Soft Tissue Sarcoma (approximately 3%) (Fig. 3a). We identified missense mutations as the primary mutation type of the LIPT2 gene, along with the R125L alteration in the BPL_LplA_LipB domain (Fig. 3b). Figure 3c depicts the R125L alteration in the 3D structure of the LIPT2 protein. Supplementary Fig. 4d presents the mutation count of LIPT2 in different cancers. We also explored the expression differences of LIPT2 gene in different mutation groups and observed significant differences in 17 types of tumors (Supplementary Fig. 4a). Furthermore, LIPT2 gene mutation groups exhibited poorer OS, DSS, and PFS (Supplementary Fig. 4c).

Due to the close correlation between LIPT2 mutation and the prognosis of GBMLGG patients (Supplementary Table 2), we explored the mutation landscape of LIPT2 in GBMLGG. The results showed that the mutation frequency distribution of several oncogenes (such as IDH1 and ATRX) and tumor suppressor genes (such as PTEN) was uneven among the group with high expression of LIPT2 and the group with low LIPT2 expression (Supplementary Fig. 4b). We also evaluated the correlation between the expression of LIPT2 and MMR genes in pan-cancer. It was found that LIPT2 expression in almost all pan-cancer samples was significantly positively pertinent to the expression of MMR genes, indicating that LIPT2 may promote cancer cell growth through positive regulation of MMR gene expression (Fig. 3d).

### Promoter methylation of LIPT2 in pan-cancer

**Figure 4:**
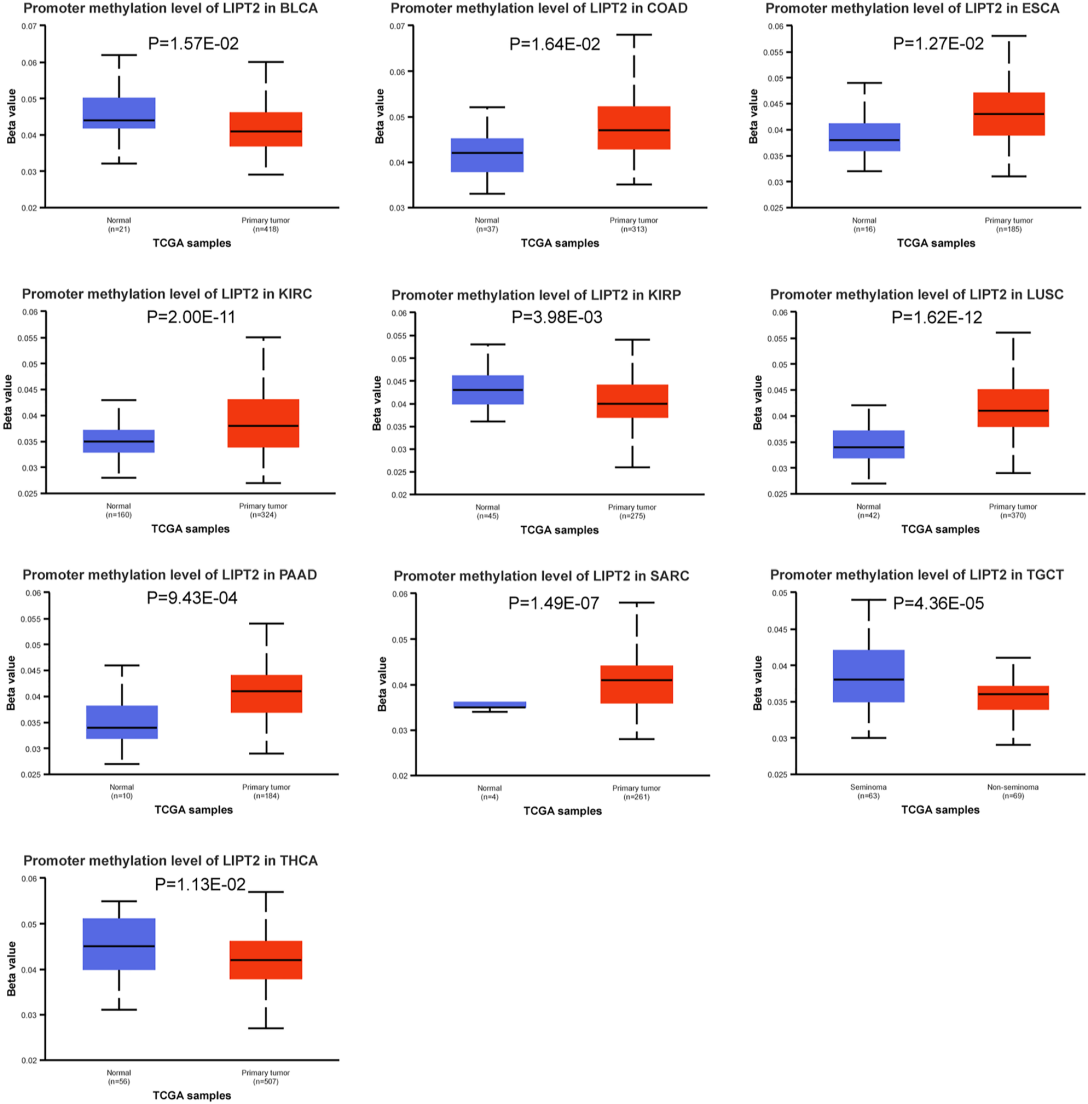
Comparison of the promoter methylation levels of LIPT2 between cancerous tissues and normal tissues using UALCAN, indicating significant differences in cancer (p < 0.05).

It has been proven that promoter methylation is engaged in tumorigenesis and progression^11^. We compared the methylation levels of LIPT2 between pan-cancer tissues and paired normal tissues using UALCAN. It was found that the level of LIPT2 promoter methylation was obviously increased in Colon adenocarcinoma (COAD), Esophageal carcinoma (ESCA), KIRC, Lung squamous cell carcinoma (LUSC), Pancreatic adenocarcinoma (PAAD), and Sarcoma (SARC) tissues compared to normal tissues, while they were significantly lower in BLCA, KIRP, THCA, and Testicular Germ Cell Tumors (TGCT) tissues (Fig. 4). Moreover, we resolved the association of LIPT2 expression in pan-cancer to 44 marker genes for three RNA methylation modifications (m1A, m5C, m6A). The findings indicated a remarkable positive correlation between the two in most cancers. However, in a few cancers, they showed a significant negative correlation (Supplementary Fig. 5). These results suggest that promoter methylation may mediate the transcriptional expression of LIPT2 and affect tumor progression.

### LIPT2 is associated with cancer immune infiltration

**Figure 5:**
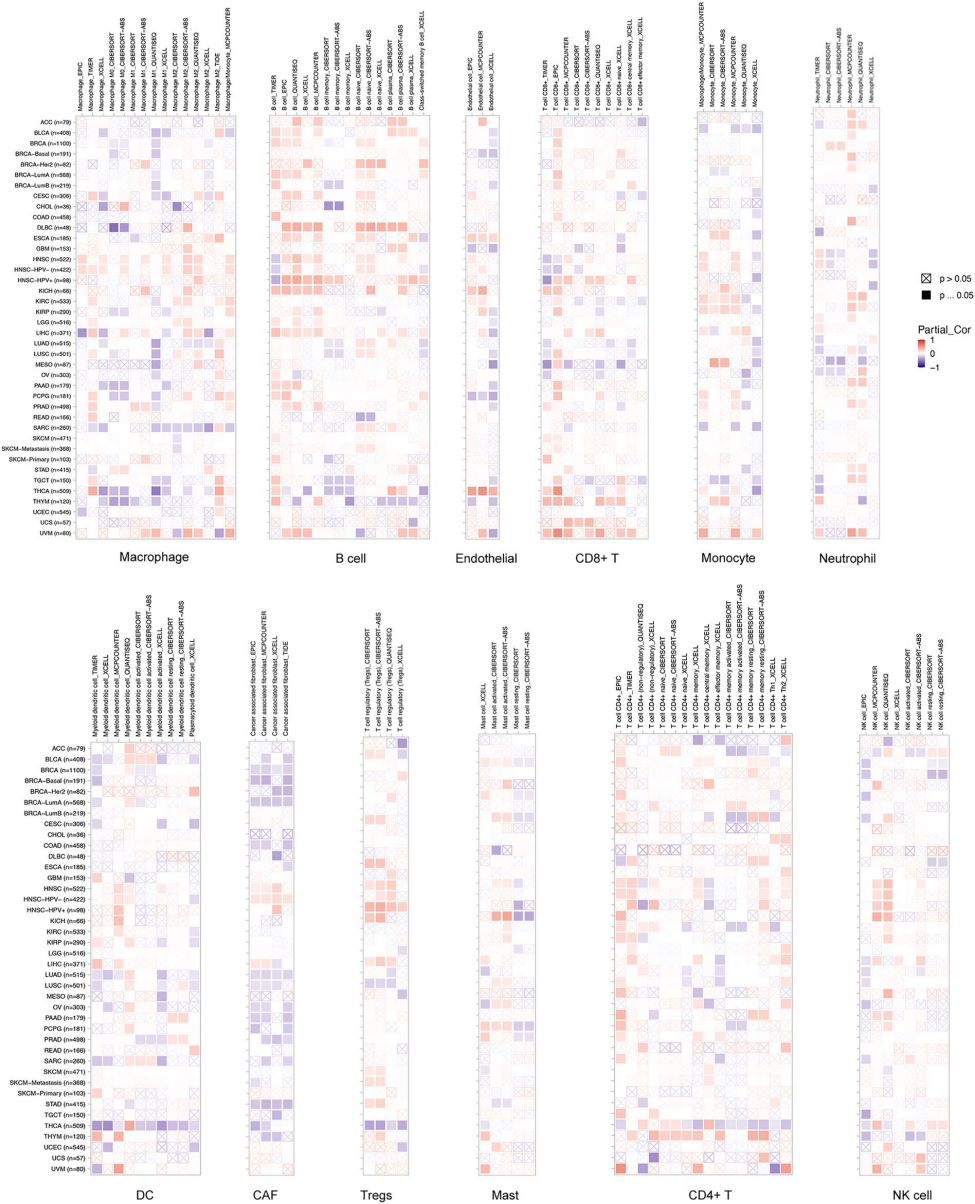
Correlation analysis between LIPT2 expression and immune cell infiltration in pan-cancer using TIMER2.0.

Recent studies have shown a close relationship between immune infiltration and cancer progression^12,13^. In the TIMER2 database, the association between LIPT2 expression and immune cell infiltration in cancer was evaluated using various algorithms (TIMER^14^, EPIC^15^, QUANTISEQ^16^, XCELL^17^, MCPCOUNTER^18^, CIBERSORT^19^, and CIBERSORT-ABS). As shown in Fig. 5, in most cancers, LIPT2 expression was negatively linked to cancer-associated fibroblasts (CAFs) infiltration levels, especially in Breast invasive carcinoma (BRCA), LUAD, and Stomach adenocarcinoma (STAD), while it was positively correlated with B-cell infiltration values, such as BLCA, BRCA, Lymphoid Neoplasm Diffuse Large B-cell Lymphoma (DLBC), KICH. Furthermore, in Head and Neck squamous cell carcinoma (HNSC), LIPT2 expression showed a positive correlation with regulatory T cells (Tregs) and macrophage infiltration, and in Uveal Melanoma (UVM), it exhibited a positive correlation with monocyte, neutrophil, and CD8+ T cell infiltration, but negatively correlated with Tregs and dendritic cell (DC) infiltration in THCA. Additionally, In ESCA and THCA, LIPT2 expression displayed a positive correlation with endothelial cell infiltration, whereas in PCPG, BRCA, GBM, and thymoma (THYM), it showed an inverse correlation with endothelial cell infiltration. However, LIPT2 expression showed no significant correlation with CD4+ T cells, NK cells, and mast cell infiltration. Next, we further evaluated the relationship between LIPT2 expression and cancer immune infiltration using the EstimateScore, ImmuneScore, and StromalScore. The results demonstrated that LIPT2 expression was negatively correlated with ImmuneScore, EstimateScore and StromalScore in most cancers (Supplementary Fig. 6a). In conclusion, the above results demonstrate the significant importance of LIPT2 in tumor cell immune infiltration.

### Expression of LIPT2 and immune response to pan-cancer immunotherapy

**Figure 6:**
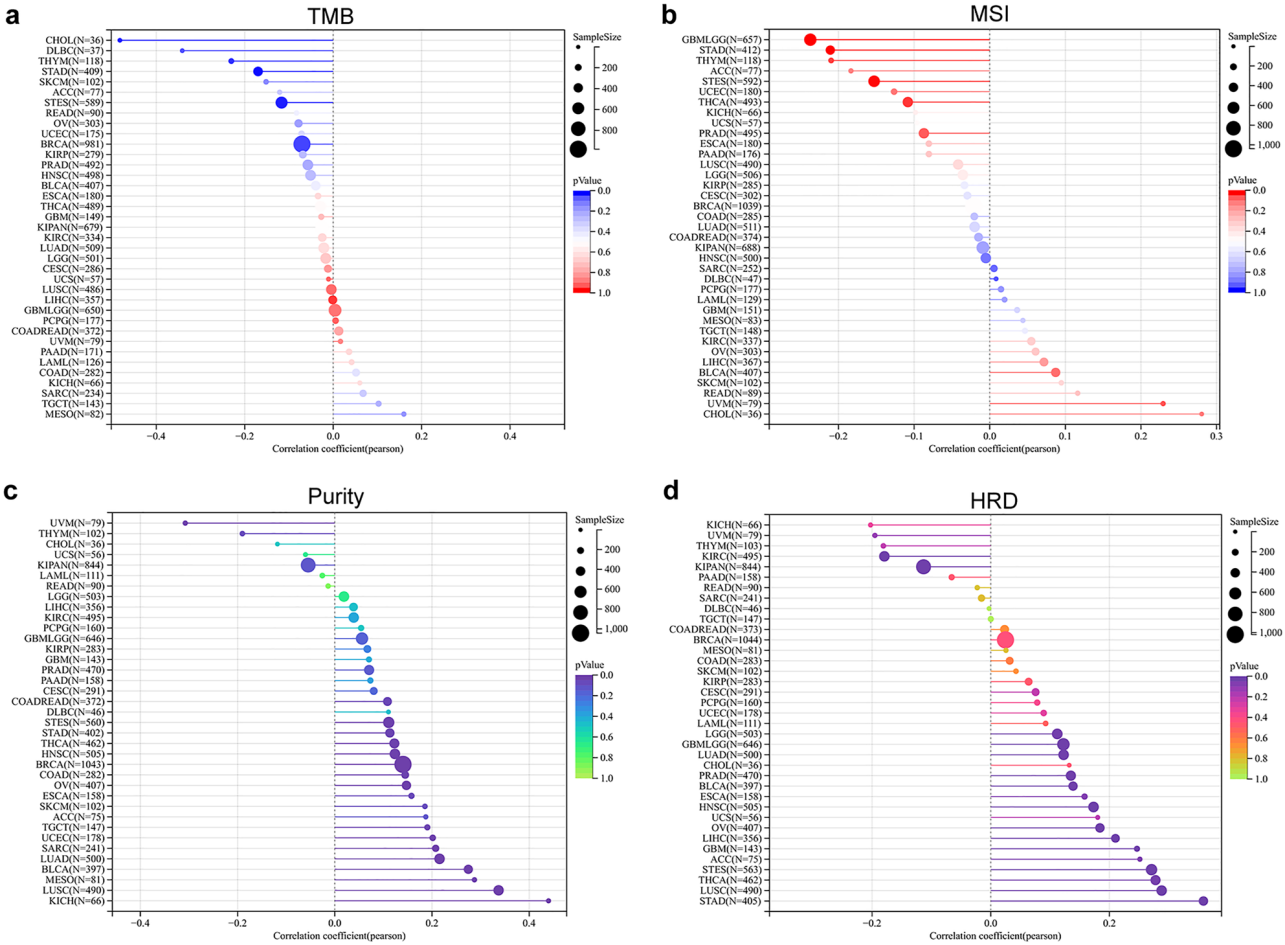
Prediction of immune therapy response in pan-cancer based on LIPT2. (**a–d**) Visualization of the correlation between LIPT2 expression and TMB (**a**), MSI (**b**), purity (**c**), HRD (**d**).

We analyzed the association between LIPT2 and immune regulatory genes (including chemokines, receptors, MHC, immunoinhibitors and immunostimulators) (Supplementary Fig. 7). In UVM and GBMLGG, most immune regulatory genes are positively correlated with LIPT2 expression levels, while in THCA they are mostly negatively correlated. Since tumor mutational burden (TMB) and microsatellite instability (MSI) are key factors in predicting the efficacy of immunotherapy, we assessed the correlation between LIPT2 expression and pan-cancer TMB and MSI. We noticed an inverse correlation between LIPT2 expression and TMB in BRCA, Stomach and Esophageal carcinoma (STES), STAD, THYM, CHOL, and DLBC (Fig. 6a), and an inverse correlation between LIPT2 expression and MSI in GBMLGG, STES, STAD, THYM, and THCA, while a positive correlation was found in UVM (Fig. 6b). Tumor purity is known to affect the efficiency of immune checkpoint inhibitor therapy, and we found a significant positive correlation between LIPT2 expression and tumor purity in 17 types of tumors, particularly in KICH and LUSC. However, in UVM, a significant negative correlation was observed (Fig. 6c). Furthermore, HRD status is a crucial metric for various tumor treatment regimens and prognosis. We observed a significant correlation between LIPT2 expression and HRD status in six types of tumors, with a positive correlation in STES and STAD, and a negative correlation in GBMLGG, BRCA, THYM, and KICH (Fig. 6d). Additionally, we assessed the predictive effect of LIPT2 on cancer immunotherapy response using the ROC Plotter database (Supplementary Fig. 6b). The findings illustrated that LIPT2 was strongly expressed in cancers that responded to any anti PD-L1 therapy, with an AUC value of 0.577 for 5-year recurrence-free survival (RFS). It was lowly expressed in patients who responded to anti CTLA-4 therapy, with an AUC of 0.645 for 5-year RFS. These results suggest that LIPT2 may function as a prognostic indicator of the effectiveness of immune therapy in corresponding cancers.

### Single-cell level expression of LIPT2

**Figure 7:**
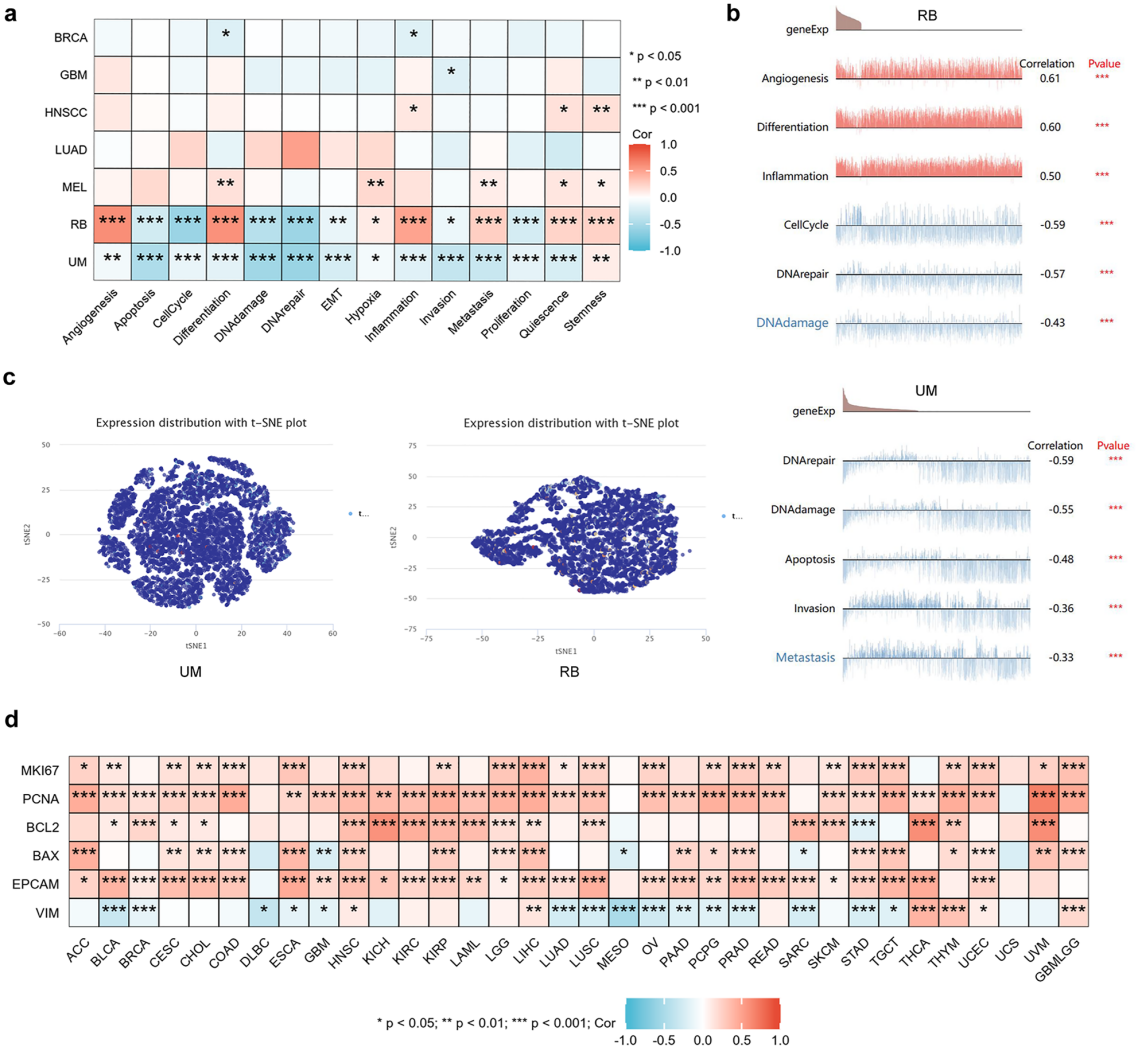
Expression of LIPT2 at the single-cell level. (**a,b**) The CancerSEA website analyzed the correlation between LIPT2 expression at the single-cell level and different functional states of tumor cells. (**c**) T-SNE map of LIPT2 expression in RB and UM. (**d**) Correlation between LIPT2 expression levels and cancer cell proliferation, apoptosis, and EMT marker genes.

We resolved the connection between LIPT2 and 14 functional states of diverse cancers. The findings revealed that LIPT2 expression was in a positive correlation with angiogenesis, differentiation, and inflammation in retinoblastoma (RB), and negatively correlated with DNA damage, DNA repair, and cell cycle. Additionally, the expression of LIPT2 was negatively correlated with cell apoptosis, DNA damage, DNA repair, invasion, and metastasis in uveal melanoma (UM) (Fig. 7a). The correlation between LIPT2 expression and functional states in different single-cell datasets also confirmed the above results (Fig. 7b). The T-SNE plot displayed the expression profiles of LIPT2 at the single-cell level in RB and UM (Fig. 7c). Furthermore, we investigated the impact of LIPT2 expression levels on cancer cell proliferation, apoptosis, and epithelial-mesenchymal transition (EMT). MKI67 and PCNA were markers for cell proliferation, BCL2 was a confirmed apoptosis suppressor, BAX was an apoptosis promoter, EPCAM was a well-known epithelial cell marker, and VIM was a mesenchymal cell marker. The outcomes showed that in the majority of cancer cases, LIPT2 expression was eminently positively correlated with MKI67, PCNA, BCL2, BAX, and EPCAM, while showing a significant negative correlation with VIM (Fig. 7d). This indicates that LIPT2 expression levels may regulate the biological behavior of cancer cells.

### Functional analysis of LIPT2 in cancer

**Figure 8:**
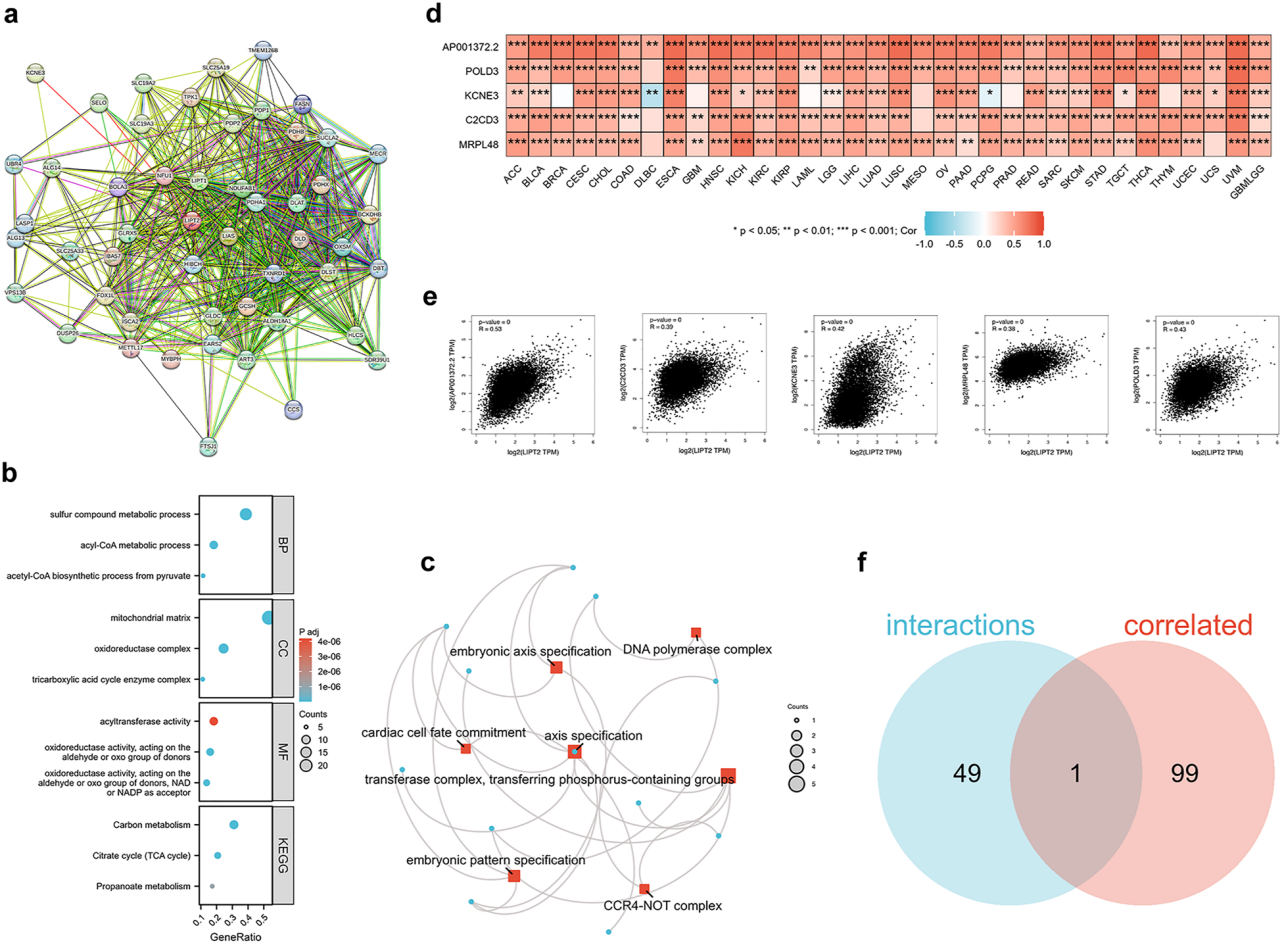
Functional enrichment analysis. (**a**) STRING tool displays the interaction network of 50 experimentally validated LIPT2 binding proteins. (**b**) GO and KEGG enrichment analysis of LIPT2 binding proteins. (**c**) Enrichment analysis of the top 100 LIPT2 expression-related genes obtained from GEPIA2. (**d,e**) Heatmap (**d**) and scatter plot (**e**) showing the correlation between LIPT2 expression and five genes (AP001372.2, POLD3, KCNE3, C2CD3, MRPL48) in pan-cancer. (**f**) Cross-analysis of LIPT2 binding proteins and related genes.

To further investigate the possible molecular mechanisms of LIPT2 in cancer occurrence and development, we performed enrichment analysis on proteins interacting with LIPT2 and LIPT2-related genes. We obtained 50 experimentally validated LIPT2 binding proteins using the STRING tool, and Fig. 8a reveals the interconnectedness of these proteins within the network. GO and KEGG enrichment analysis of these proteins indicated their participation in biological processes (BP) such as sulfur compound metabolic and acyl-CoA metabolic, cellular components (CC) including mitochondrial matrix and oxidoreductase complex, and molecular functions (MF) such as acyltransferase activity and oxidoreductase activity (Fig. 8b). These proteins were primarily associated with Carbon metabolism and the TCA cycle pathway (Fig. 8b). Using the GEPIA2 tool in combination with TCGA tumor expression data, we identified the top 100 genes associated with LIPT2 expression (Supplementary Table 3). Enrichment analysis demonstrated that these genes were mostly tied to transferase complex, transferring phosphorus-containing groups, axis specification, embryonic pattern specification, and embryonic axis specification (Fig. 8c). In most tumors, LIPT2 expression levels were positively correlated with the expression levels of AP001372.2, POLD3, KCNE3, C2CD3, and MRPL48 (Fig. 8d,e). Cross-analysis of LIPT2 binding proteins and LIPT2 expression-related genes revealed a common member, namely KCNE3 (Fig. 8f).

## Discussion

Emerging research suggests that cuproptosis is affiliated with the occurrence, progression, and prognosis of diverse human cancers^20–23^. In this analysis, we comprehensively evaluated the value of the cuproptosis-related gene LIPT2 in pan-cancer through multiple bioinformatics platforms. Based on the pan-cancer expression profiles from the TGCA, GTEx, and TARGET databases, LIPT2 showed significantly high expression in 26 tumor tissues, including GBMLGG, compared to normal human tissues. It was significantly downregulated in three tumor types and no significant statistical expression differences were found in five tumor types. This implies that LIPT2 is likely a new cancer biomarker. Furthermore, the expression level of LIPT2 influences the prognosis of cancer patients. In GBM, GBMLGG, LGG, KICH, WT, and PCPG, LIPT2 is a risk factor with high expression predicting poor prognosis for patients. However, in some tumors, LIPT2 plays a protective role, showing a good prognosis with high expression, including KIPAN, KIRC, KIRP, CHOL, and OV. It is worth mentioning that LIPT2 expression is closely correlated with GBMLGG patients’ OS, DSS, and PFI indicators. The ROC curve suggests that LIPT2 expression has a diagnostic value for GBMLGG with an accuracy of 85.5%. These findings indicate that LIPT2 has strong potential as a prognostic biomarker for cancer patients, especially in GBMLGG, providing direction for future research. However, the biological function of LIPT2 in GBMLGG still needs further experimental verification, which is also the drawback of this study.

The occurrence of tumors is often closely associated with gene mutations^24^. Mutations in MMR genes impair the integrity of the normal cell genome and lead to genomic instability^25^. DNA methylation is a significant epigenetic modification that can affect gene expression and cellular function^26^. MMR gene mutations and DNA methylation changes are strongly tied to the tumor progression^27^. In this study, we observed LIPT2 gene mutations in various tumors, and the LIPT2 mutant group had poorer OS, DSS, and PFS. In almost all cancers, MMR gene mutations were significantly positively correlated with LIPT2 expression. Furthermore, in COAD, ESCA, KIRC, LUSC, PAAD, and SARC tissues, LIPT2 methylation levels were markedly higher than in normal tissues, while in BLCA, KIRP, THCA, and TGCT tissues, they were significantly lower. These results indicate that LIPT2 exerts an essential function in tumor occurrence at both the genetic and epigenetic levels. Single-cell sequencing results suggest that LIPT2 may regulate multiple biological actions of cancer, such as DNA damage repair, angiogenesis, cell cycle, cell apoptosis, invasion, and metastasis. Functional enrichment analysis reveals the potential molecular pathways of LIPT2 in cancer initiation and progression.

Immunocytes play a crucial role in recognizing cancer cells and regulating tumor growth^28^. B cells are most well-known for their production of antibodies, such as IgE, IgG, IgA, and IgM^29^. CAFs exert a pivotal function in inducing cancer cell growth and metastasis^30^. Tregs maintain immune homeostasis through various pathways^31^. In this research, we identified that LIPT2 expression is strongly linked to the infiltration of diverse immune cells in cancer, including CAFs, B cells, Tregs, monocytes, macrophages, neutrophils, CD8+ T cells, dendritic cells (DCs), and endothelial cells. Immune checkpoint blockade therapies (including anti-PD-1, anti-PD-L1, and anti-CTLA4) have become hot topics in cancer immunotherapy in the near past, and their therapeutic value in cancer has been recognized^32,33^, changing the landscape of cancer treatment^34,35^. The results of this study suggest that LIPT2 has a good predictive effect on the response to cancer immunotherapy. In cancers responding to any anti-PD-L1 therapy, the AUC for predicting 5-year RFS using LIPT2 was 0.577. In patients responding to anti-CTLA-4 therapy, the AUC for 5-year RFS reached 0.645. Therefore, we believe that LIPT2 can serve as a tumor immune-related biomarker with potential clinical value in cancer treatment.

In summary, our study systematically analyzed the expression differences, prognosis, methylation, genetic alterations, immune regulation, and immune therapy of the cuproptosis-related gene LIPT2 in pan-cancer utilizing diverse bioinformatics techniques. We also investigated the expression and potential molecular regulatory mechanisms of LIPT2 at the single-cell level. This provides new directions for the prognosis and immune therapy of cancer in the future.

## Materials and methods

### Expression analysis

Standardized pan-cancer datasets, TCGA and TCGA_GTEx, were obtained from the UCSC database (https://xenabrowser.net/)^36^. The expression data for the gene ENSG00000175536 (LIPT2) was extracted and underwent log2(x + 0.001) transformation. Cancer types with less than three samples were excluded, resulting in expression data for 26 and 34 cancer types, respectively. Using the “Multi-dataset merging and batch effect correction tool” module in the Sangerbox 3.0 online tool (http://vip.sangerbox.com/)^37^, which integrates the ComBat function to merge the data and perform batch correction. Differential expression between normal and tumor samples in each tumor was calculated using R software (version 3.6.4) and analyzed for significance using non-paired Wilcoxon Rank Sum and Signed Rank Tests, with p < 0.05 considered statistically significant. Obtained LIPT2 total protein expression level from CPTAC dataset in the “Proteomics” module of UALCAN website (http://ualcan.path.uab.edu/analysis-prot.html)^38,39^. Retrieved LIPT2 immunohistochemistry images from HPA database (https://www.proteinatlas.org/)^40^. Furthermore, the HPA database provides expression levels of LIPT2 mRNA and protein in healthy human tissues, as well as expression levels of LIPT2 mRNA in cancer cell lines.

### Survival analysis

We obtained high-quality prognostic data for TCGA from a study published in Cell (An Integrated TCGA Pan-Cancer Clinical Data Resource to Drive High-Quality Survival Outcome Analytics)^41^. Additionally, we supplemented this with TCGA_GTEx follow-up data from UCSC. We excluded samples with follow-up times less than 30 days and cancer types with fewer than 10 samples, resulting in expression data for 44, 38, 32, and 38 cancer types, along with corresponding samples for Overall Survival (OS), Disease-specific Survival (DSS), Disease-free Interval (DFI), and Progression-free Interval (PFI) data. We used the R package “maxstat” to compute the optimal cutoff value for LIPT2, dividing patients into high and low groups. Furthermore, we employed the R package “survival” to establish a Cox proportional hazards regression model and plotted Kaplan–Meier curves, evaluating prognostic differences significantly using the logrank test, where p < 0.05 was considered statistically significant. Additionally, we analyzed the correlation between LIPT2 expression and clinical features of GBMLGG, and plotted receiver operating characteristic (ROC) curves. Based on the Cox regression analysis of OS, we constructed a nomogram and performed calibration curves to evaluate the predictive accuracy at 1, 3, and 5 years.

### Genetic mutation analysis

We analyzed the mutation types, frequency, count, sites, and three-dimensional (3D) structure of the LIPT2 protein in the “TCGA Pan-Cancer Atlas Study” cohort through the cBioPortal tool (https://www.cbioportal.org/) in the “Cancer Type Summary” and “Mutation” modules^42^. In the “Comparison” module, the clinical prognosis of all TCGA cancer types with or without LIPT2 gene alterations was also analyzed, including OS, DSS, disease-free survival (DFS), and progression-free survival (PFS). In addition, we downloaded the Copy Number Variation (CNV) dataset of all TCGA samples processed by GISTIC software from GDC (https://portal.gdc.cancer.gov/). We extracted expression data of LIPT2 gene and DNA mismatch repair (MMR) genes (MLH1, MLH3, MSH2, MSH3, MSH6, PMS1, and PMS2) from the TCGA dataset and performed log2(x + 0.001) transformation. Further, we generated partial correlation (cor) and p-value through Pearson rank correlation test. The data is visualized in the form of a heatmap.

### Methylation analysis

The methylation levels of LIPT2 in tumor tissues and normal tissues were analyzed through the UALCAN website using TCGA dataset. Using the “RNA modification gene analysis” module of Sangerbox 3.0 online tool^37^, The expression data of LIPT2 gene and 44 marker genes of three types of RNA modifications (m1A(10), m5C(13), m6A(21)) were extracted from the TCGA_GTEx dataset in each sample and log2(x + 0.001) transformed. A heatmap was generated through Pearson correlation analysis.

### Single cell analysis

We explored the relevance of the correlation of LIPT2 expression at the single-cell level with various functional states of tumor cells using the CancerSEA database (http://biocc.hrbmu.edu.cn/CancerSEA/)^43^, and displayed the expression profile of LIPT2 in single cells through T-SNE plots.

### Enrichment analysis

We applied the STRING website (https://string-db.org/)^44^ to obtain a network analysis of experimentally determined LIPT2-binding proteins. The top 100 LIPT2 expression-related genes were accessed from the TCGA dataset using the GEPIA 2.0 (http://gepia2.cancer-pku.cn)^45^ tool, with Pearson correlation analysis performed on the selected genes. At the same time, the R package (clusterProfiler 4.4.4) was used to conduct Gene Ontology (GO) and Kyoto Encyclopedia of Genes and Genomes (KEGG) analysis on the above molecules.

### Immune assessment

Based on the “Immune-Gene” module in the TIMER2.0 (http://timer.cistrome.org/) database^46^, various algorithms were used to evaluate the association between LIPT2 expression and immune cell infiltration in all TCGA tumors. p values and partial correlation (cor) values were obtained through purity-adjusted Spearman rank correlation tests. The results were presented in the form of a heatmap. The expression data of LIPT2 gene and 150 genes related to five immune pathways (chemokine (41), receptor (18), MHC (21), Immunoinhibitor (24), Immunostimulator (46)) were extracted from the TCGA_GTEx dataset, and then log2(x + 0.001) transformation was applied. The Pearson correlation between LIPT2 and the marker genes of the five immune pathways was calculated in each sample. In addition, we also extracted the gene expression profiles of each tumor from the TCGA_GTEx dataset, mapped the expression profiles to GeneSymbol, and used the R software package “ESTIMATE” to calculate stromal, immune, and ESTIMATE scores for patients in each tumor based on LIPT2 expression. Finally, we obtained immune infiltration scores for 10,180 tumor samples in 44 tumor types and calculated the Pearson correlation between LIPT2 gene and immune infiltration scores in each tumor. We downloaded the Simple Nucleotide Variation dataset of all TCGA samples processed by the MuTect2 software from GDC, and used the R package “maftools” to calculate the Tumor mutation burden (TMB) for each tumor. We obtained the Microsatellite instability (MSI) scores for each tumor from a previous study (Landscape of Microsatellite Instability Across 39 Cancer Types)^47^. We also obtained the purity and homologous recombination deficiency (HRD) data for each tumor from a published study (The Immune Landscape of Cancer)^48^. We integrated the TMB, MSI, purity, HRD, and gene expression data for the samples, and calculated their Pearson correlation in each type of tumor. The prediction of LIPT2 on the efficacy of cancer immunotherapy was obtained using the ROC Plotter (https://www.rocplot.org/) online tool^49^.

### Supplementary Information

Supplementary Figure S1.

Supplementary Figure S2.

Supplementary Figure S3.

Supplementary Figure S4.

Supplementary Figure S5.

Supplementary Figure S6.

Supplementary Figure S7.

Supplementary Table S1.

Supplementary Table S2.

Supplementary Table S3.

## Data Availability

The data provided by this study can be found in the following online tools. UCSC (https://xenabrowser.net/), Sangerbox3.0 (http://vip.sangerbox.com/), UALCAN (http://ualcan.path.uab.edu/analysis-prot.html), HPA (https://www.proteinatlas.org/), cBioPortal (https://www.cbioportal.org/), GDC (https://portal.gdc.cancer.gov/), CancerSEA (http://biocc.hrbmu.edu.cn/CancerSEA/), STRING (https://string-db.org/), GEPIA2.0 (http://gepia2.cancer-pku.cn), TIMER2.0 (http://timer.cistrome.org/), ROC Plotter (https://www.rocplot.org/).
