# Supplementary figures and images for: Comprehensive analysis of the potential biological significance of cuproptosis-related gene LIPT2 in pan-cancer prognosis and immunotherapy
Source: Sci Rep. 2023 Dec 21;13:22910. doi: 10.1038/s41598-023-50039-x (PMC10739704; doi:10.1038/s41598-023-50039-x)

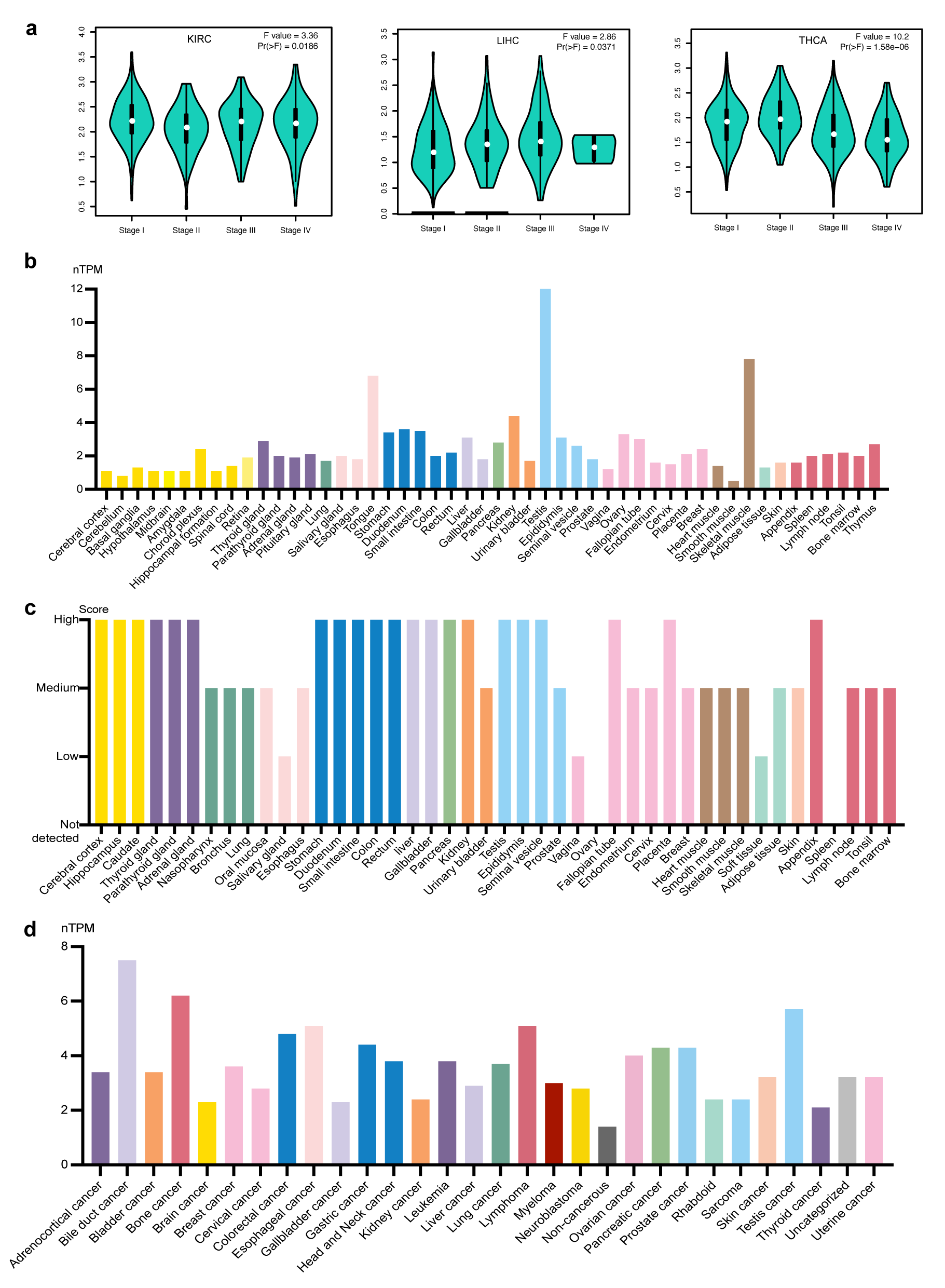

Supplement: Supplementary file 1 — Supplementary Figure S1. [file 41598_2023_50039_MOESM1_ESM.tif]

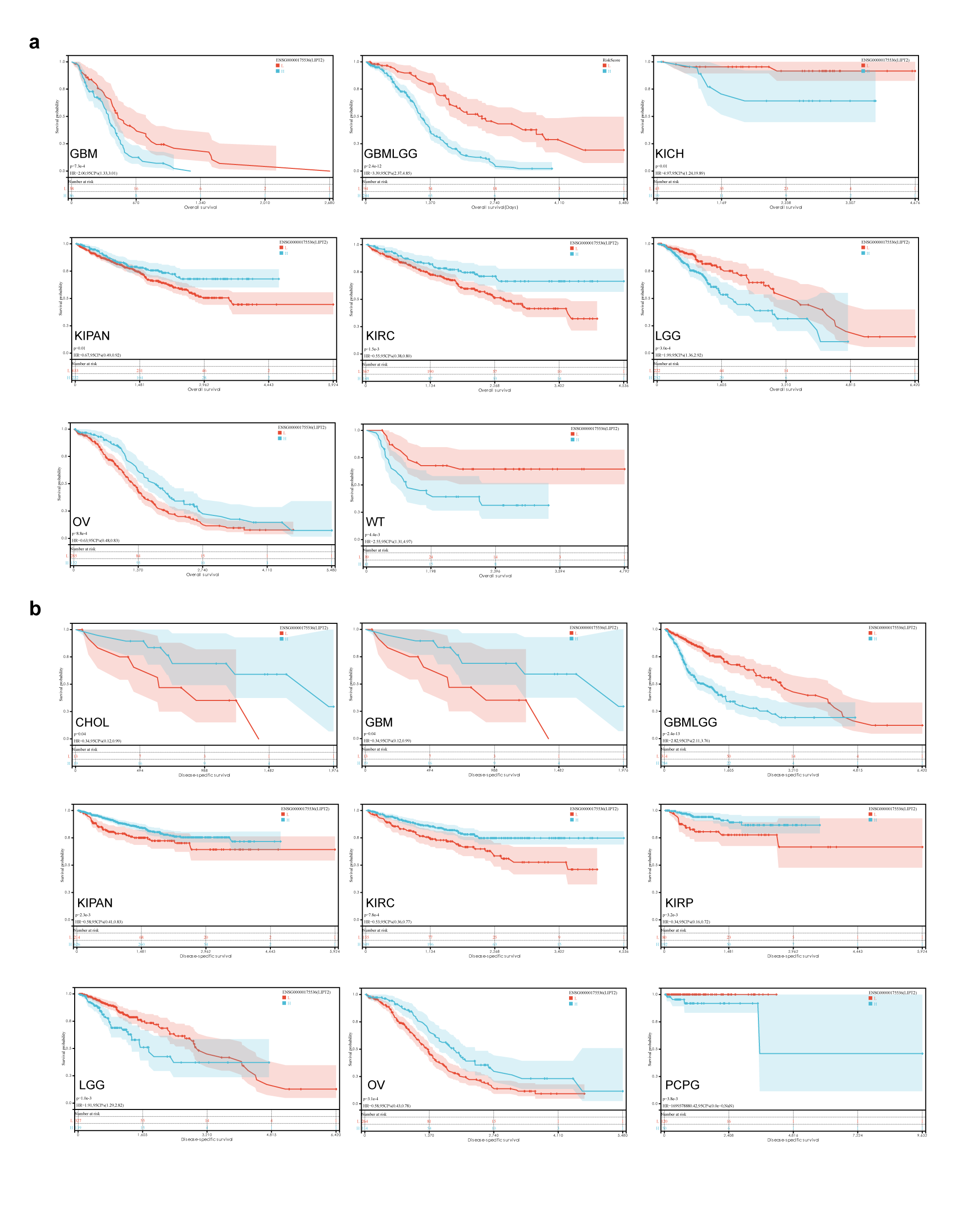

Supplement: Supplementary file 2 — Supplementary Figure S2. [file 41598_2023_50039_MOESM2_ESM.tif]

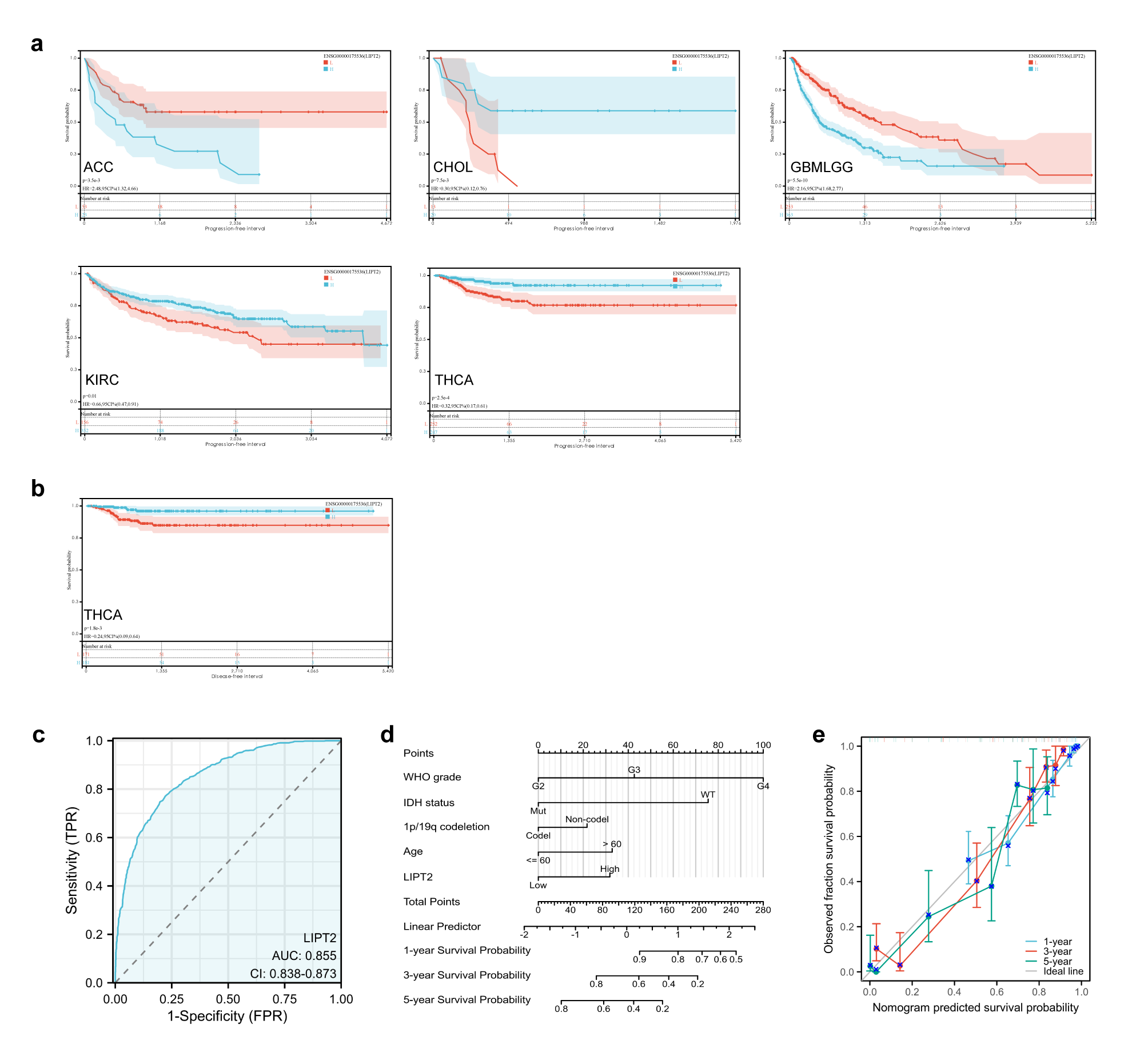

Supplement: Supplementary file 3 — Supplementary Figure S3. [file 41598_2023_50039_MOESM3_ESM.tif]

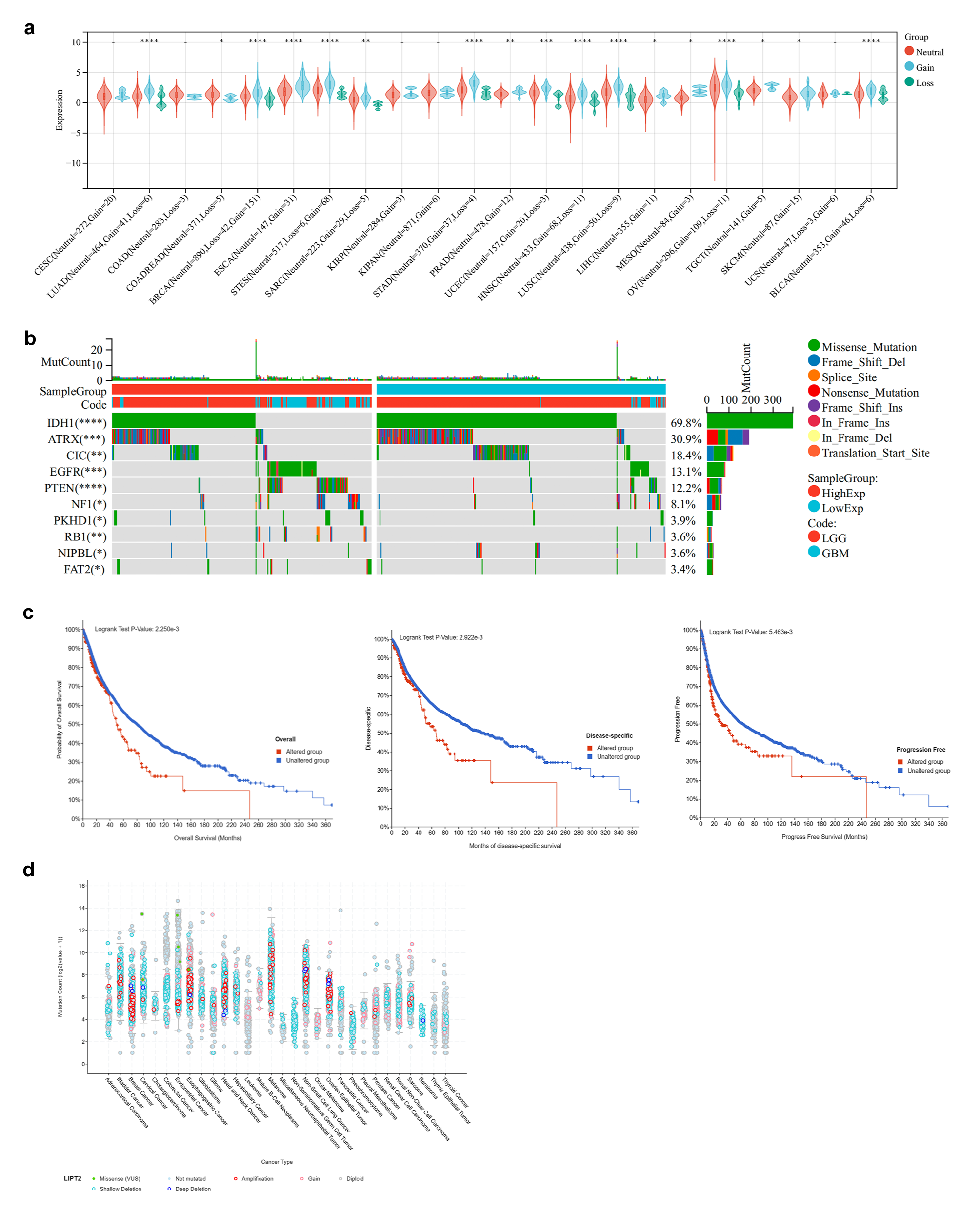

Supplement: Supplementary file 4 — Supplementary Figure S4. [file 41598_2023_50039_MOESM4_ESM.tif]

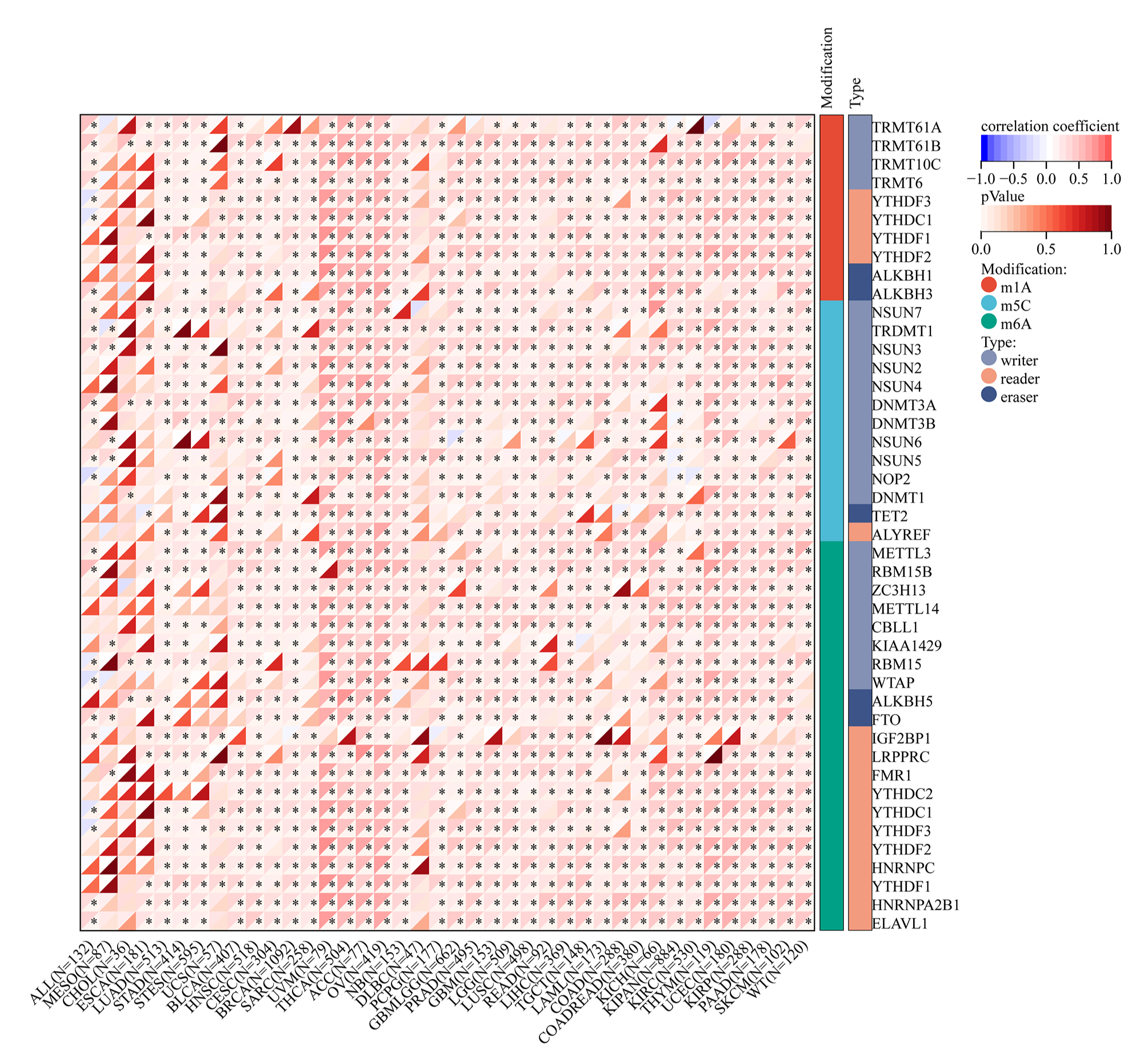

Supplement: Supplementary file 5 — Supplementary Figure S5. [file 41598_2023_50039_MOESM5_ESM.tif]

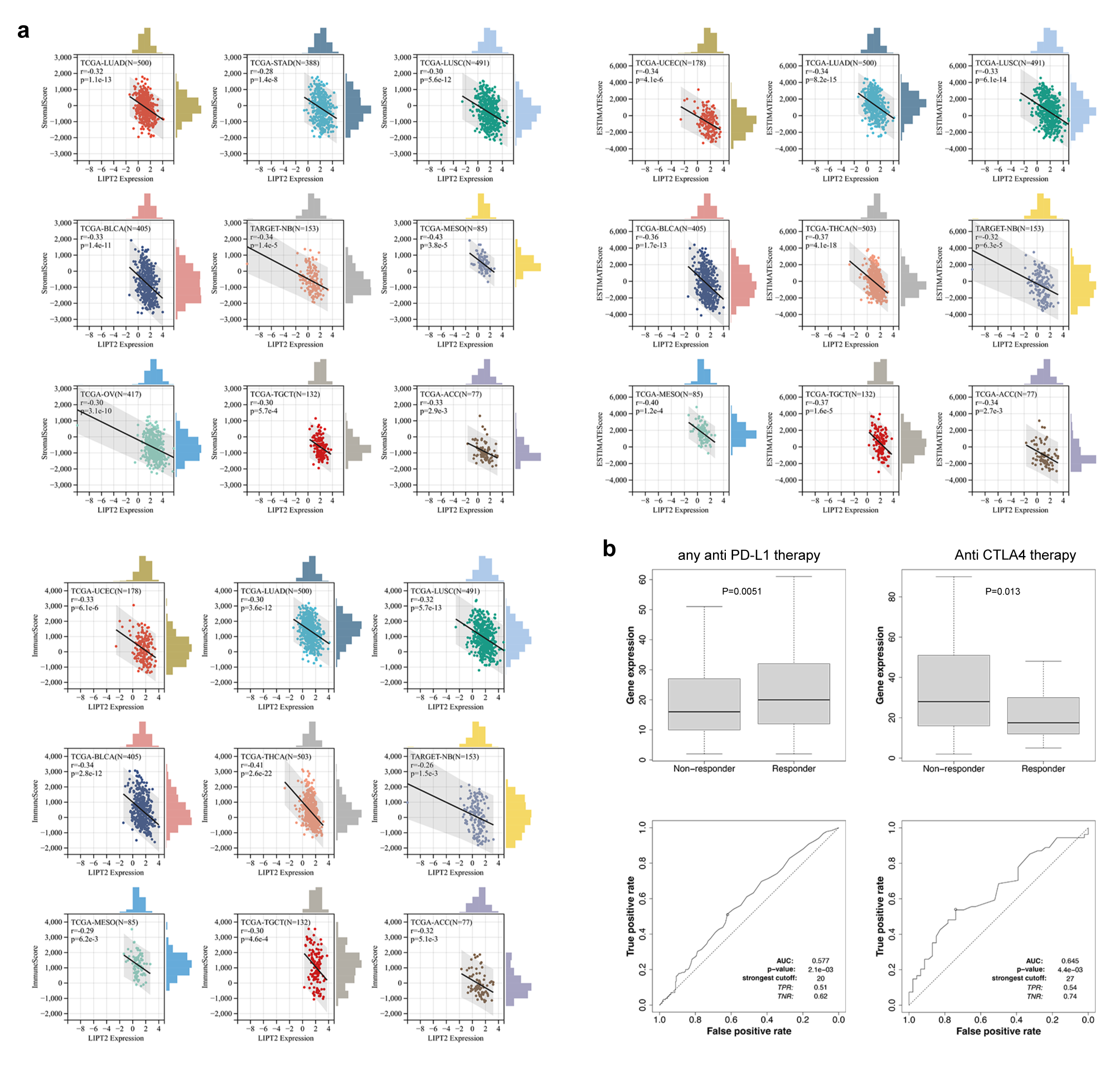

Supplement: Supplementary file 6 — Supplementary Figure S6. [file 41598_2023_50039_MOESM6_ESM.tif]

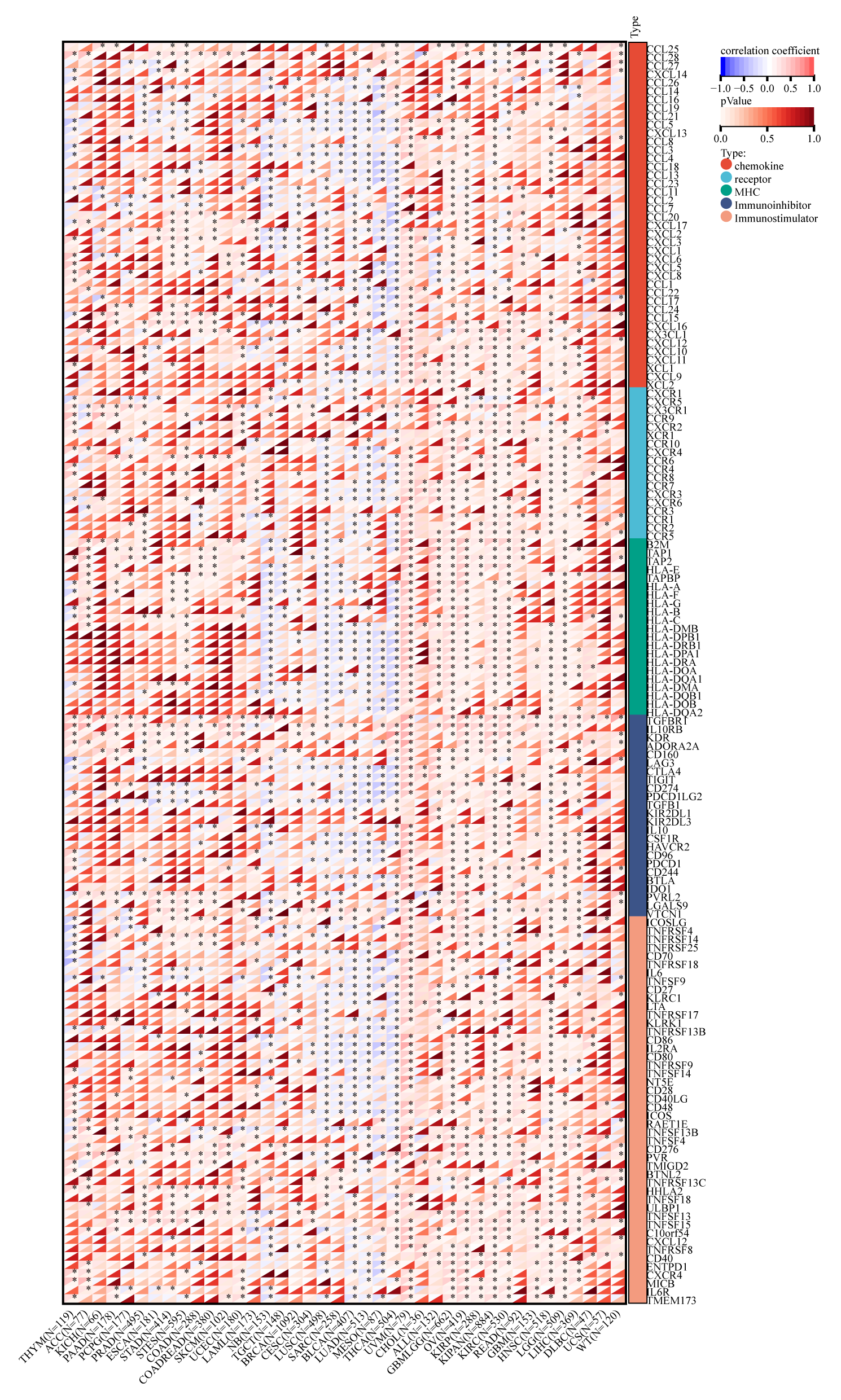

Supplement: Supplementary file 7 — Supplementary Figure S7. [file 41598_2023_50039_MOESM7_ESM.tif]
