# Supplementary material for: Comprehensive analysis of the potential biological significance of cuproptosis-related gene LIPT2 in pan-cancer prognosis and immunotherapy
Source: Sci Rep. 2023 Dec 21;13:22910. doi: 10.1038/s41598-023-50039-x (PMC10739704; doi:10.1038/s41598-023-50039-x)
Supplement: Supplementary file 8 — Supplementary Table S1. [file 41598_2023_50039_MOESM8_ESM.docx]

**TABLE 1.** Correlation between LIPT2 expression and the clinicopathological features of the GBMLGG cases.

| Characteristics | Low expression of LIPT2 | High expression of LIPT2 | P value |
| --- | --- | --- | --- |
| n | 349 | 350 |  |
| WHO grade, n (%) |  |  | < 0.001 |
| G2 | 135 (21.2%) | 89 (14%) |  |
| G3 | 129 (20.3%) | 116 (18.2%) |  |
| G4 | 45 (7.1%) | 123 (19.3%) |  |
| IDH status, n (%) |  |  | < 0.001 |
| WT | 89 (12.9%) | 157 (22.8%) |  |
| Mut | 257 (37.3%) | 186 (27%) |  |
| 1p/19q codeletion, n (%) |  |  | < 0.001 |
| Non-codel | 241 (34.8%) | 279 (40.3%) |  |
| Codel | 105 (15.2%) | 67 (9.7%) |  |
| Gender, n (%) |  |  | 0.035 |
| Female | 135 (19.3%) | 163 (23.3%) |  |
| Male | 214 (30.6%) | 187 (26.8%) |  |
| Race, n (%) |  |  | 0.447 |
| Asian | 6 (0.9%) | 7 (1%) |  |
| Black or African American | 13 (1.9%) | 20 (2.9%) |  |
| White | 323 (47.1%) | 317 (46.2%) |  |
| Age, n (%) |  |  | 0.004 |
| <= 60 | 293 (41.9%) | 263 (37.6%) |  |
| > 60 | 56 (8%) | 87 (12.4%) |  |
| Histological type, n (%) |  |  | < 0.001 |
| Astrocytoma | 113 (16.2%) | 83 (11.9%) |  |
| Oligoastrocytoma | 80 (11.4%) | 55 (7.9%) |  |
| Oligodendroglioma | 111 (15.9%) | 89 (12.7%) |  |
| Glioblastoma | 45 (6.4%) | 123 (17.6%) |  |
