# Supplementary material for: Comprehensive analysis of the potential biological significance of cuproptosis-related gene LIPT2 in pan-cancer prognosis and immunotherapy
Source: Sci Rep. 2023 Dec 21;13:22910. doi: 10.1038/s41598-023-50039-x (PMC10739704; doi:10.1038/s41598-023-50039-x)
Supplement: Supplementary file 9 — Supplementary Table S2. [file 41598_2023_50039_MOESM9_ESM.docx]

| Characteristics | Total(N) | Univariate analysis | |  | Multivariate analysis | |
| --- | --- | --- | --- | --- | --- | --- |
|  |  | Hazard ratio (95% CI) | P value |  | Hazard ratio (95% CI) | P value |
| WHO grade | 636 |  | **< 0.001** |  |  |  |
| G2 | 223 | Reference |  |  | Reference |  |
| G3 | 245 | 2.967 (1.986 - 4.433) | **< 0.001** |  | 1.953 (1.270 - 3.002) | **0.002** |
| G4 | 168 | 18.600 (12.448 - 27.794) | **< 0.001** |  | 4.799 (2.861 - 8.048) | **< 0.001** |
| IDH status | 688 |  | **< 0.001** |  |  |  |
| WT | 246 | Reference |  |  | Reference |  |
| Mut | 442 | 0.116 (0.089 - 0.151) | **< 0.001** |  | 0.306 (0.206 - 0.456) | **< 0.001** |
| 1p/19q codeletion | 691 |  | **< 0.001** |  |  |  |
| Non-codel | 520 | Reference |  |  | Reference |  |
| Codel | 171 | 0.225 (0.147 - 0.346) | **< 0.001** |  | 0.712 (0.430 - 1.177) | 0.185 |
| Age | 698 |  | **< 0.001** |  |  |  |
| <= 60 | 555 | Reference |  |  | Reference |  |
| > 60 | 143 | 4.696 (3.620 - 6.093) | **< 0.001** |  | 1.674 (1.229 - 2.280) | **0.001** |
| LIPT2 | 698 |  | **< 0.001** |  |  |  |
| Low | 349 | Reference |  |  | Reference |  |
| High | 349 | 2.073 (1.621 - 2.651) | **< 0.001** |  | 1.647 (1.256 - 2.160) | **< 0.001** |

**TABLE 2.** Univariate and multivariate Cox regression analyses of the clinical characteristics associated with overall survival in TCGA-GBMLGG.
